# Morroniside Improves Diabetic Osteoporosis via the AGE/RAGE/Wnt/β‐Catenin Signaling Pathway

**DOI:** 10.1002/kjm2.70063

**Published:** 2025-06-26

**Authors:** Lu Yang, Kai Wang, Zhao‐Hui Zeng, Hai‐En Zhao, Li‐Min Bai

**Affiliations:** ^1^ Department of Endocrinology The Second Affiliated Hospital of Air Force Medical University Xi'an China; ^2^ Department of Orthopaedics The Second Affiliated Hospital of Air Force Medical University Xi'an China; ^3^ Department of Geriatrics The Second Affiliated Hospital of Air Force Medical University Xi'an China

**Keywords:** AGE/RAGE axis, diabetic osteoporosis, morroniside, osteoblasts, Wnt/β‐catenin signaling pathway

## Abstract

Morroniside has been shown to possess various pharmacological activities, including anti‐inflammatory and antioxidative effects. This study investigates the potential mechanisms by which morroniside ameliorates diabetic osteoporosis (DOP). In vivo experiments were conducted to evaluate biochemical parameters in rats under different treatment conditions. Bone tissues underwent HE staining, and bone mineral density (BMD) along with key bone metabolism markers (β‐CTX, OC, SOST) were measured. The activities of antioxidant enzymes (SOD, GPX, CAT), oxidative stress indicators (MDA), and levels of inflammatory factors (MCP‐1, IL‐6, IL‐1, TNF‐α) were also assessed. Western blot was used to analyze the expression of proteins associated with AGE/RAGE signaling and the Wnt/β‐catenin pathway. Morroniside increased trabecular bone quantity and quality, upregulated the bone formation markers OC and SOST, downregulated the bone resorption marker β‐CTX, and significantly increased BMD. Additionally, it modulated the systemic metabolic status by reducing fasting blood glucose (FBG), glycated hemoglobin (HbA1c), free fatty acids (FFA), triglycerides (TG), and total cholesterol (TC). Further research indicated that morroniside inhibited AGE/RAGE signaling, mitigated oxidative stress and inflammatory responses, and enhanced Wnt/β‐catenin pathway activity, thereby promoting osteoblast proliferation, differentiation, and mineralization. The introduction of DKK1 significantly attenuated these beneficial effects of morroniside, confirming its protective role through activation of the Wnt/β‐catenin pathway. Morroniside exerts beneficial effects on bone metabolism and quality in DOP rats by regulating the AGEs/RAGE/Wnt/β‐catenin signaling pathway, while also alleviating oxidative stress and inflammatory responses. These findings provide novel insights and potential therapeutic targets for the treatment of DOP.

## 1 Introduction

Diabetic osteoporosis (DOP) is a common complication in patients with diabetes, characterized by reduced bone mineral density (BMD), bone microstructure degeneration, and increased fracture risk [1]. As the global incidence of diabetes continues to rise, DOP has become a significant public health concern. Research indicates that hyperglycemia impairs skeletal health through multiple mechanisms, including heightened oxidative stress, exacerbated inflammatory responses, and accumulation of advanced glycation end products (AGEs) [2]. AGEs are complex compounds formed through non‐enzymatic reactions between reducing sugars and proteins, lipids, or nucleic acids [3]. In a hyperglycemic environment, AGEs are excessively produced and accumulated, subsequently activating their specific receptor, RAGE. This activation triggers downstream signaling pathways such as NF‐κB and MAPK, leading to chronic inflammation and cellular dysfunction [4]. Osteoblasts, the primary cell type responsible for bone matrix production, play a critical role in bone formation and maintenance. Inhibiting diabetes‐induced osteoblast apoptosis can significantly mitigate bone loss [5]. AGEs can induce osteoblast apoptosis; it has been reported that AGEs inhibit bone formation in vitro by weakening osteoblast lineage differentiation, inducing osteoblast apoptosis, suppressing the expression of osteoblast‐specific transcription factors, and reducing mineralization [6].

Recent studies have demonstrated that certain natural products and their extracts exhibit preventive and therapeutic effects against diabetes and its complications [7, 8], Flavonoids derived from licorice roots possess anti‐hyperglycemic properties, mitigating diabetes‐related complications, including those affecting bone metabolism [9, 10]. These compounds inhibit glucose elevation in vivo, reduce the production of AGEs, and lower parathyroid hormone levels, thereby improving calcium homeostasis in bones. Additionally, they enhance osteoblast activity, suppress osteoclast function, maintain alkaline phosphatase (ALP) activity, and reduce adenosine triphosphate (ATP) levels in bone cells. A review study [11] reported that red sage leaf extracts significantly decreased blood glucose and insulin‐related enzymatic activity in diabetic mouse models while increasing the levels of superoxide dismutase (SOD), catalase (CAT), and glutathione.

Morroniside (Mor), the major bioactive component of *Cornus officinalis* , exhibits diverse biological activities, including anti‐apoptotic, anti‐oxidative, and anti‐ischemic effects [12]. Studies have demonstrated its protective effects against AGEs‐induced injury in rat glomerular mesangial cells (GMCs), which manifests as restoration of damaged mesangial cell morphology, reduction in extracellular matrix (ECM) secretion, and inhibition of AGEs‐induced expression of RAGE, p38MAPK, NF‐κB, and TGF‐β in GMCs—with protective efficacy increasing in a concentration‐dependent manner. Additionally, Morroniside mitigates high‐glucose (HG)‐mediated dysfunction in bone marrow mesenchymal stem cells (BMSCs) and promotes HG‐induced osteogenic differentiation of BMSCs in vitro, partly through inhibition of the AGE/RAGE signaling pathway and activation of glyoxalase 1 (Glo1) [13]. In follicular outer root sheath cells (ORSCs), Morroniside treatment enhances cell proliferation and migration while upregulating the expression of Wnt10b and β‐catenin; notably, treatment with the Wnt/β‐catenin signaling inhibitor DKK1 partially reverses the promoting effects of Morroniside on ORSC proliferation and migration [14]. Additionally, research by Sun et al. has found that in ischemic stroke, Morroniside promotes neurogenesis and aids brain recovery by activating the Wnt/β‐catenin signaling pathway, suggesting its potential value in the treatment of stroke [15]. Despite these findings, its specific effects on DOP and the interplay between AGE/RAGE and Wnt/β‐catenin pathways in this context remain unclear.

The Wnt/β‐catenin signaling pathway is a critical molecular network for maintaining bone homeostasis, participating in the entire developmental process from stem cells to mature osteocytes [16]. Under normal conditions, when Wnt ligands bind to their membrane surface receptors, intracellular β‐catenin protein stabilizes, translocates to the nucleus, and co‐activates target gene expression with members of the TCF/LEF transcription factor family, including osteogenic genes such as RUNX2 and OCN [17]. However, in DOP, the activity of the Wnt/β‐catenin pathway is suppressed due to negative regulation from AGEs, leading to decreased bone formation, increased bone resorption, and ultimately bone loss with reduced bone strength [18]. The interaction between the AGE/RAGE axis and the Wnt/β‐catenin signaling pathway in DOP pathogenesis remains unclear.

Therefore, this study hypothesizes that morroniside may be a potential therapeutic agent for DOP, with the AGE/RAGE and Wnt/β‐catenin signaling pathways potentially serving as targets for its action. Building on the research background outlined above, this project aims to elucidate the therapeutic effects of morroniside in a DOP rat model, investigate its impact on AGE‐induced osteoblast differentiation and apoptosis, and analyze whether the AGE/RAGE and Wnt/β‐catenin signaling pathways are involved in its mechanism of action.

## 2 Materials and Methods

### 2.1 Animal Models

This study utilized 70 male Sprague–Dawley (SD) rats aged 6–7 weeks, weighing approximately 200–250 g (purchased from Beijing Vital River Laboratory Animal Technology Co. Ltd., license number: SCXK (Jing) 2019–0010). All animals were housed in a specific‐pathogen‐free (SPF) environment with ad libitum access to food and water, maintained under a 12‐h light/dark cycle. A DOP rat model was induced using a combination of a high‐fat diet and streptozotocin (STZ). After a one‐week acclimatization period, rats were fasted for 12–16 h and divided into two groups: the modeling group (*n* = 60), which received a high‐fat diet for 8 weeks followed by intraperitoneal injections of low‐dose STZ (35 mg/kg, dissolved in 0.1 mol/L sodium citrate buffer, pH 4.5), and continued on the high‐fat diet for an additional week; and the control group (*n* = 10), which was fed standard chow and injected with sodium citrate buffer at corresponding time points. Tail vein blood samples were collected to measure fasting plasma glucose (FPG), with FPG ≥ 16.7 mmol/L indicating successful model establishment. The experimental protocol was approved by the Ethics Committee of the Second Affiliated Hospital of Air Force Medical University and conducted in accordance with national regulations for laboratory animal care and use.

The successfully modeled DOP rats were randomly divided into five groups: the DOP group, low‐dose morroniside group (DOP + L‐Mor), medium‐dose morroniside group (DOP + M‐Mor), high‐dose morroniside group (DOP + H‐Mor), and medium‐dose morroniside plus DKK1 group (DOP + M‐Mor + DKK1). The control group (*n* = 10) consisted of age‐matched control rats maintained under standard conditions. Rats in the DOP + L‐Mor, DOP + M‐Mor, and DOP+ H‐Mor groups were orally administered morroniside (catalog number: S5465, Selleck Chemical, USA) at doses of 30, 90, and 270 mg/kg, respectively. Rats in the DOP + M‐Mor + DKK1 group received 90 mg/kg morroniside and 6 μg/kg recombinant DKK1 protein via oral gavage. Treatment lasted for 12 weeks, during which body weight was monitored weekly, and fasting blood glucose (FBG) was measured using a Roche Accu‐Chek Performa glucometer. Fasting insulin (FINS) levels were determined using ELISA kits (Mercodia, catalog numbers 10‐1149‐01 and 10‐1102‐01), and the insulin sensitivity index was calculated accordingly.

### 2.2 Cell Culture and Treatment

Newborn SD rat calvariae were aseptically harvested, stripped of connective tissue, and cut into 2–5 mm^2^ pieces. These fragments were digested in 0.25% trypsin–EDTA, and the digestion was terminated by adding fetal bovine serum (FBS). The supernatant was then discarded, and the remaining tissue fragments were seeded in α‐MEM containing 1% penicillin–streptomycin and 10% FBS. Cultures were maintained at 37°C in a humidified atmosphere with 5% CO_2_. The medium was changed every 2 days, and experiments were performed using cells at passage 3 or 4.

Osteoblasts were divided into four groups: Control, AGE, AGE + morroniside (AGE + Mor), and AGE + morroniside + DKK1 (AGE + Mor + DKK1). Cells in the AGE group were treated with 100 μg/mL AGE‐BSA (BIOVISION, San Francisco, USA) for 48 h. Cells in the AGE + Mor group were treated with 100 μg/mL AGE and 100 μM morroniside for 48 h. Cells in the AGE + Mor + DKK1 group were treated with 100 μg/mL AGE‐BSA, 100 μM morroniside, and 4 μg/L DKK1 for 48 h. The concentration of morroniside (100 μM) was determined based on previous studies demonstrating its protective effects in osteoblasts under high‐glucose or AGE‐induced stress [19] and preliminary dose–response tests in our laboratory. DKK1 (4 μg/L) was used according to preliminary experiments for Wnt pathway inhibition in osteoblastic cells.

#### 2.2.1 Cell Proliferation and Apoptosis Assays

Cell proliferation was assessed using the MTT assay. Osteoblasts (5 × 10^3^ cells/well) were seeded into 96‐well plates and incubated with different treatments for 48 h, followed by MTT incubation for 4 h. Absorbance was measured at 572 nm after solubilizing formazan crystals with dimethyl sulfoxide (DMSO). Apoptosis was detected using an Annexin V‐FITC/PI apoptosis detection kit. Cells (5 × 10^5^ cells/well) were seeded in 6‐well plates, treated with drugs for 48 h, washed with PBS, and analyzed using a Cytomics FC 500 flow cytometer.

#### 2.2.2 Alkaline Phosphatase (ALP) Activity Assay

After drug treatment, ALP staining and activity assays were performed. Cells were fixed in 4% paraformaldehyde, stained using the BCIP/NBT alkaline phosphatase color development kit (Beyotime, catalog number P0202), lysed in RIPA buffer, and ALP activity was measured at 520 nm.

#### 2.2.3 Reactive Oxygen Species (ROS) Measurement

Intracellular ROS generation was measured using the DCFH‐DA probe by flow cytometry. Cells were incubated with 10 μM DCFH‐DA for 30 min in the dark, and cell fluorescence was measured using a flow cytometer.

#### 2.2.4 Alizarin Red Staining

Osteoblasts (1 × 10^5^ cells/well) were seeded in six‐well plates containing osteogenic differentiation induction medium. After 21 days, the cells were fixed in 4% paraformaldehyde, stained with 0.1% alizarin red (pH 8.3) for 30 min at room temperature. Mineralized nodules were stained with 0.1% Alizarin Red S (ARS) solution (pH 4.2) for 30 min, washed with distilled water, and visualized under an optical microscope (Olympus, Tokyo, Japan). For quantification, cell‐bound ARS was dissolved in 10% acetic acid by heating at 85°C for 10 min, and the absorbance was measured at 562 nm using a microplate reader (BioTek, Winooski, USA) to determine calcium deposition levels.

### 2.3 Histological Analysis

Rat tibiae were fixed in 4% paraformaldehyde (Solarbio, catalog number P1110), decalcified, embedded, sectioned, and stained with hematoxylin–eosin (HE, Solarbio, catalog number G1020) and Masson's trichrome (Solarbio, catalog number G1330).

Micro‐CT scanning (NMC‐200, Pingsheng Medical Technology) was used to evaluate trabecular bone parameters such as bone volume fraction (BV/TV), trabecular thickness (Tb.Th), trabecular number (Tb.N), and trabecular separation (Tb.Sp). Micro‐CT scanning (NMC‐200, Pingsheng Medical Technology) was performed using the following parameters: X‐ray voltage 80 kV, current 0.06 mA, pixel resolution 0.051 × 0.051 × 0.051 mm, and filtered back‐projection (FDK) reconstruction algorithm. The scanning was conducted without additional filter settings.

### 2.4 ELISA Detection

Serum β‐CTX levels were measured using a Rat β‐Crosslaps ELISA Kit (R&D Systems, catalog number: BMS207/3), OC levels with the Rat Osteocalcin ELISA Kit (Elabscience, catalog number: E‐EL‐R0206c), and SOST levels with the Mouse/Rat Sclerostin ELISA Kit (R&D Systems, catalog number: DS004). For lipid and glucose metabolism, TG, TC, FPG, and HbA1c levels were measured using ELISA kits from Elabscience (TG: E‐EL‐K0344c, TC: E‐EL‐K0343c, FPG: E‐EL‐K0338c, HbA1c: E‐EL‐K0340c). Samples were diluted and added to pre‐coated wells, incubated at room temperature for 2 h, washed, and biotinylated antibodies were added. After a further hour of incubation, horseradish peroxidase‐conjugated streptavidin was added, followed by TMB substrate solution. Reactions were stopped, and absorbance was read at 450 nm.

### 2.5 Oxidative Stress and Inflammatory Factor Detection

Serum superoxide dismutase (SOD, Beyotime, Shanghai, China, catalog number: S0101), glutathione peroxidase (GPX, Beyotime, Shanghai, China, catalog number: S0053), catalase (CAT, Beyotime, Shanghai, China, catalog number: S0057), and malondialdehyde (MDA, Beyotime, Shanghai, China, catalog number: S0033) levels were measured using respective kits. Inflammatory factors MCP‐1 (Elabscience, Wuhan, China, catalog number: E‐EL‐R0023c), IL‐6 (Elabscience, Wuhan, China, catalog number: E‐EL‐R0019c), IL‐1 (Elabscience, Wuhan, China, catalog number: E‐EL‐R0002c), and TNF‐α (Elabscience, Wuhan, China, catalog number: E‐EL‐R0012c) were detected using ELISA kits. Intracellular ROS production was measured using the DHE kit (Beyotime, catalog number: S0063) via flow cytometry.

### 2.6 Immunohistochemistry

Immunohistochemical staining was performed according to standard protocols using DAB chromogen (Vector Laboratories, catalog number SK‐4100). Sections were deparaffinized, antigen retrieval was performed in pH 6.0 buffer (Solarbio, catalog number G1242), and sections were blocked with goat serum (Solarbio, catalog number AR1001). Rabbit anti‐RAGE antibody (Abcam, catalog number ab3611; 1:200 dilution) was applied overnight at 4°C. The next day, biotinylated goat anti‐rabbit IgG (Jackson ImmunoResearch, catalog number 111‐035‐003; 1:200 dilution) was added and incubated for 1 h at room temperature. DAB was used for visualization, followed by counterstaining with hematoxylin, dehydration, clearing, and mounting.

### 2.7 BMD Measurement

Appendicular skeletal mineral content was assessed using dual‐energy X‐ray absorptiometry (DXA; Lunar iNSiGHT VET, OsteoSys, Seoul). Three‐dimensional BMD parameters were acquired for total body (excluding cranial structures), with scan analyses conducted by blinded operators using Encore v16.1 software (GE Healthcare).

### 2.8 Western Blotting

Total proteins were extracted from tissues or cells using RIPA lysis buffer (Beyotime, catalog number P0013B) supplemented with protease inhibitor cocktail (Roche, catalog number 11836170001) and quantified using a BCA protein assay kit (Beyotime, catalog number P0010).

Proteins were separated by electrophoresis on 4%–12% Bis‐Tris gels (Invitrogen, catalog number NP0335BOX) and transferred to PVDF membranes (Millipore, catalog number IPVH00010). Membranes were blocked with 5% skim milk and incubated overnight at 4°C with primary antibodies against β‐catenin (Cell Signaling Technology, catalog number 8480; 1:1000), Wnt‐3a (Santa Cruz Biotechnology, catalog number sc‐13866; 1:500), ALP, OCN, OPN, RUNX2 (Abcam, catalog numbers ab83236, ab93876, ab8446, ab76956; all 1:1000), and GAPDH (Proteintech, catalog number 60004‐1‐Ig; 1:5000). HRP‐conjugated goat anti‐rabbit IgG (Jackson ImmunoResearch, catalog number 111‐035‐003; 1:5000) was used as the secondary antibody, and membranes were incubated with secondary antibodies for 1 h at room temperature. Chemiluminescence imaging was performed using ECL reagent (Millipore, catalog number WBKLS0500).

### 2.9 qRT‐PCR

Total RNA was extracted using TRIzol Reagent (Invitrogen, catalog number 1559601) and quantified using a NanoDrop 2000 spectrophotometer (Thermo Fisher Scientific). RNA was reverse‐transcribed into cDNA using the PrimeScript RT reagent Kit with gDNA Eraser (Takara, catalog number RR047A). Real‐time PCR was performed using SYBR Premix Ex Taq II (Takara, catalog number RR820A) on an ABI 7500 Real‐Time PCR System. Primer sequences for each gene were provided. Relative expression levels were calculated using the 2^−ΔΔCt^ method.

### 2.10 Statistical Analysis

Data are expressed as mean ± standard deviation and were analyzed using GraphPad Prism 8 software. Comparisons between two groups were performed using *t*‐tests, while multiple group comparisons were analyzed using one‐way ANOVA followed by Tukey's honestly significant difference (HSD) test for post hoc analysis. *p* < 0.05 was considered statistically significant.

## 3 Results

### 3.1 Therapeutic Effects of Morroniside on Diabetic Osteoporosis in Rats

**FIGURE 1:**
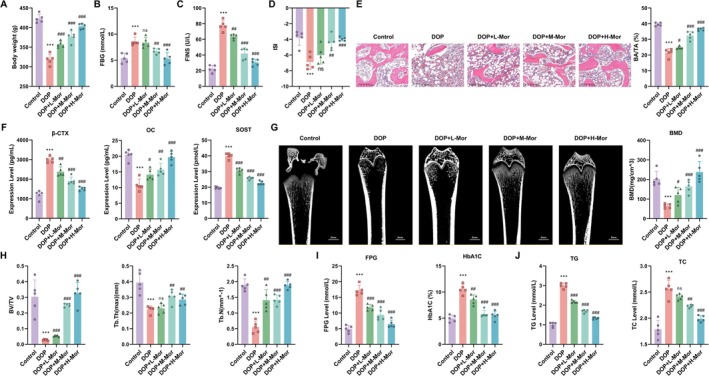
Effects of Morroniside on body weight, glycemic control, insulin sensitivity, and bone metabolism in diabetic osteoporotic rats. (A) Body weight changes in Control, DOP, and Morroniside‐treated groups (DOP + L‐Mor, DOP + M‐Mor, DOP + H‐Mor). (B) Fasting blood glucose (FBG) levels in the same groups. (C) Fasting insulin (FINS) levels and (D) Insulin sensitivity index (ISI) in the groups. (E) Histological evaluation of bone tissue by HE staining. (F) Expression of key bone metabolism markers. (G) Micro‐CT images of the right femur in Control, DOP, DOP + L‐Mor, DOP + M‐Mor, and DOP + H‐Mor groups. (H) Measurement of trabecular bone parameters (bone volume fraction (BV/TV), trabecular thickness (Tb.Th), trabecular number (Tb.N)) in the right femur of each group. (I and J) Effects of Morroniside on glycometabolism (FPG, HbA1c) and lipid metabolism (TG, TC) indicators. *n* = 5, Data are presented as mean ± SEM. *p < 0.05, **p < 0.01, ***p < 0.001 versus Control; #*p* < 0.05, ##*p* < 0.01, ###*p* < 0.001 versus DOP.

To evaluate the therapeutic potential of morroniside in DOP, we conducted in vivo experiments with five groups: the Control group, the DOP group, the low‐dose morroniside group (DOP + L‐Mor), the medium‐dose morroniside group (DOP + M‐Mor), and the high‐dose morroniside group (DOP + H‐Mor). The results showed that, compared with the Control group, rats in the DOP group exhibited significantly reduced body weight (*p* < 0.01). Treatment with different doses of morroniside increased body weight in DOP rats, most notably in the high‐dose group (Figure 1A). Additionally, FBG levels were significantly higher in the DOP group than in the Control group (*p* < 0.001), while morroniside treatment resulted in dose‐dependent reductions in FBG levels (Figure 1B). FINS levels were significantly elevated in the DOP group (*p* < 0.001), whereas morroniside‐treated groups showed significantly lower FINS levels (*p* < 0.001), with the most pronounced improvement observed in the high‐dose group (Figure 1C). The insulin sensitivity index (ISI) was significantly decreased in the DOP group (*p* < 0.001) but significantly increased in all morroniside‐treated groups (*p* < 0.001), particularly in the high‐dose group (Figure 1D). These findings indicate that morroniside can significantly improve glycemic control and insulin sensitivity in a DOP model.

Further analysis via HE staining of bone tissues revealed significant trabecular bone sparsity and structural damage in the DOP group. However, morroniside treatment significantly mitigated these pathological changes, particularly in the high‐dose group (Figure 1E). Expression of key bone metabolism markers showed elevated β‐CTX (indicative of bone resorption) and reduced levels of OC (bone formation) and SOST (regulating bone remodeling) in the DOP group. Morroniside treatment reversed these trends, decreasing β‐CTX and SOST while increasing OC (Figure 1F). For Figure 1G, micro‐CT images of the right femur were displayed. The Control group presented a normal trabecular bone structure. The DOP group showed a disrupted, less dense trabecular bone structure. In the DOP + L‐Mor, DOP + M‐Mor, and DOP + H‐Mor groups, trabecular bone structures progressively improved, with the DOP + H‐Mor group demonstrating the most significant restoration. For Figure 1H, analysis of bone volume fraction (BV/TV), trabecular thickness (Tb.Th), and trabecular number (Tb.N) showed that the DOP group had significantly lower values than the Control group. Mor treatments (low, medium, high doses) significantly reversed these parameters. The DOP + H‐Mor group exhibited the highest recovery, with notable differences from the DOP group, indicating a dose‐dependent effect of Mor on enhancing trabecular bone parameters. Collectively, morroniside not only improved BMD and bone formation marker levels but also effectively regulated glucose and lipid parameters in DOP rats (Figure 1H,I).

**FIGURE 2:**
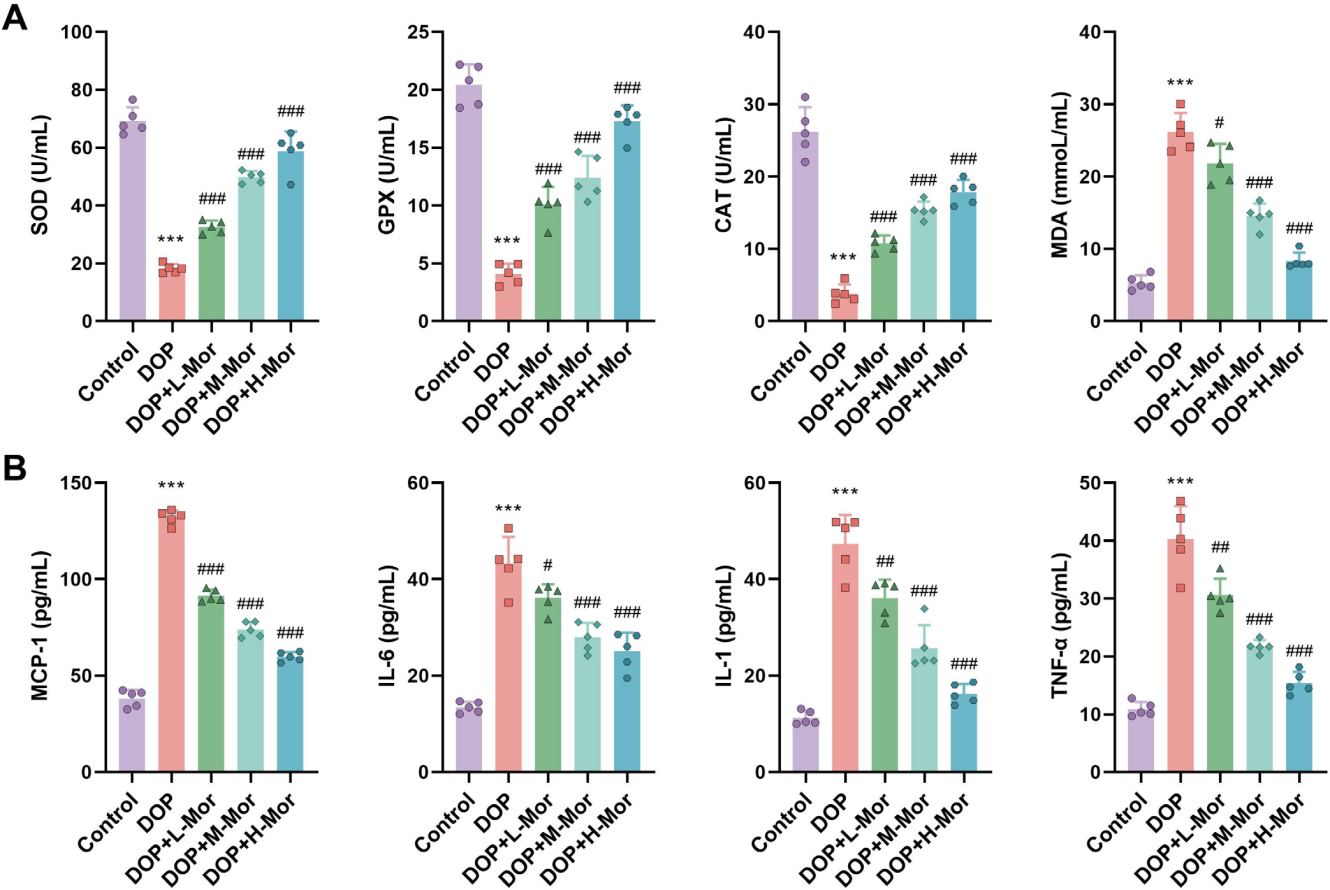
Effects of Morroniside on antioxidant enzyme activity, oxidative stress, and inflammatory cytokine levels in diabetic osteoporotic rats. (A) Activities of superoxide dismutase (SOD), glutathione peroxidase (GPX), and catalase (CAT), and (B) Malondialdehyde (MDA) levels and inflammatory cytokine levels including MCP‐1, IL‐6, IL‐1, and TNF‐α in Control, DOP, and Morroniside‐treated groups. *n* = 5, Data are presented as mean ± SEM. *p < 0.05, **p < 0.01, ***p < 0.001 versus Control; #*p* < 0.05, ##*p* < 0.01, ###*p* < 0.001 versus DOP.

Antioxidant enzyme activity, oxidative stress, and inflammatory factor levels were also evaluated. In the DOP group, activities of superoxide dismutase (SOD), glutathione peroxidase (GPX), and catalase (CAT) were significantly reduced, while malondialdehyde (MDA) levels were significantly elevated (*p* < 0.001). Morroniside treatment significantly increased SOD, GPX, and CAT activities and decreased MDA levels (*p* < 0.001), with the most significant effects observed in the high‐dose group (Figure 2A). Levels of inflammatory factors MCP‐1, IL‐6, IL‐1, and TNF‐α were significantly elevated in the DOP group (*p* < 0.001). Morroniside treatment significantly lowered these inflammatory factors (*p* < 0.001), with the greatest effect observed in the high‐dose group (Figure 2B). These results further support the potential therapeutic role of morroniside in DOP.

### 3.2 Inhibition of AGE/RAGE Signaling by Morroniside

**FIGURE 3:**
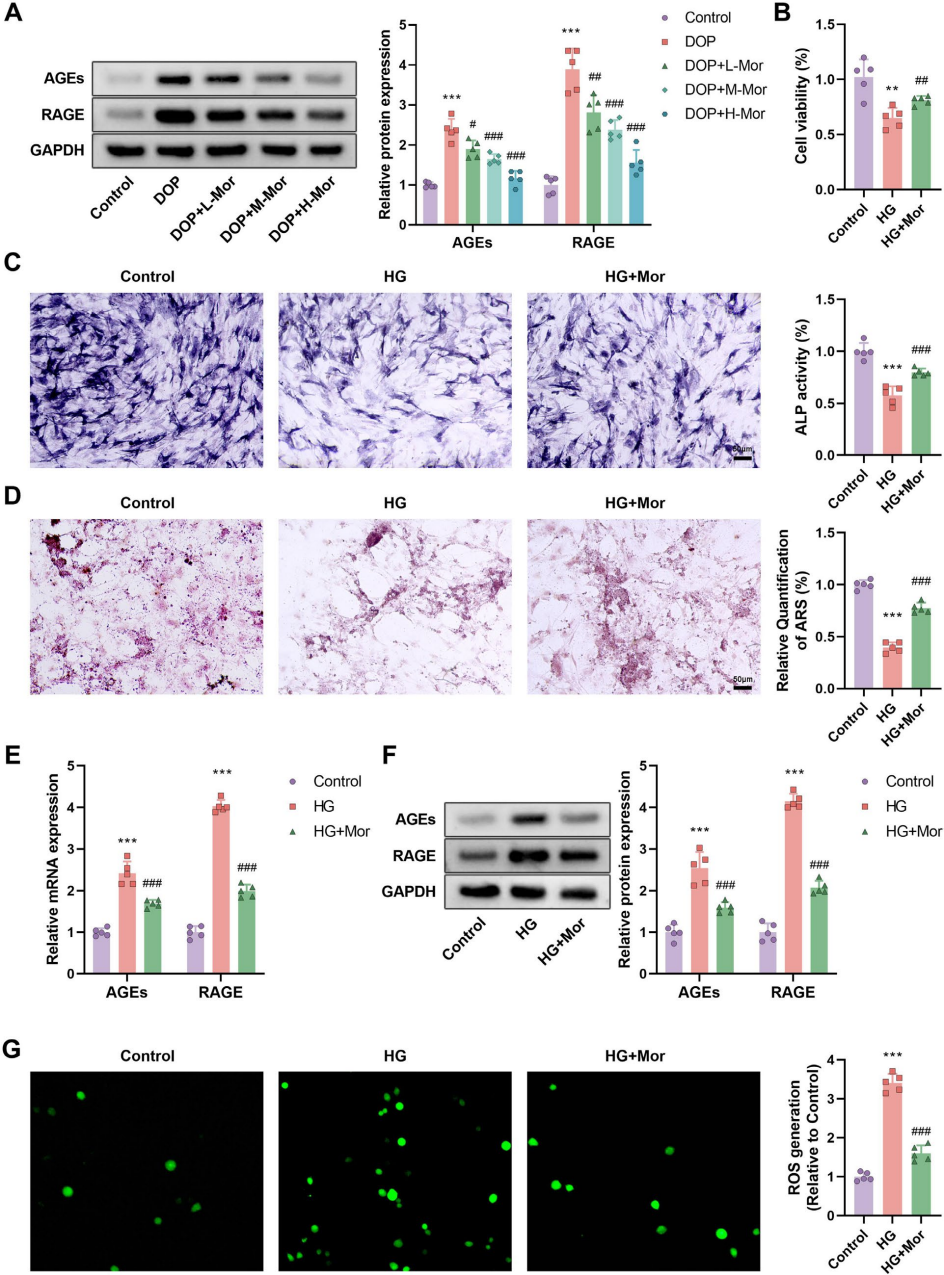
Inhibition of AGE/RAGE signaling by Morroniside in diabetic osteoporosis. (A) Western blot analysis of RAGE and AGEs protein expression in Control, DOP, and Morroniside‐treated groups. (B) MTT assay for osteoblast proliferation. (C) ALP activity and (D) Alizarin Red S (ARS) staining for osteoblast mineralization in high glucose (HG) conditions with or without Morroniside treatment. (E) mRNA expression and (F) protein expression of AGEs and RAGE under HG conditions. (G) Intracellular ROS levels measured by DHE assay. *n* = 5, data are presented as mean ± SEM. *p < 0.05, **p < 0.01, ***p < 0.001 versus Control; #*p* < 0.05, ##*p* < 0.01, ###*p* < 0.001 versus HG.

We further investigated the therapeutic potential of morroniside through inhibition of the AGE/RAGE signaling axis at both animal and cellular levels. Western blot analysis showed that protein expression of RAGE and AGEs was significantly increased in the DOP group, an effect markedly attenuated by morroniside treatment, especially in the high‐dose group (DOP + H‐Mor) (Figure 3A). MTT assays indicated that high glucose (HG) significantly reduced cell viability, an effect significantly reversed by morroniside treatment (Figure 3B). ALP activity assays demonstrated that HG significantly decreased ALP activity (*p* < 0.001), indicating inhibition of early osteoblast differentiation in a high‐glucose environment. Morroniside treatment significantly increased ALP activity (*p* < 0.001), suggesting promotion of normal osteoblast differentiation (Figure 3C). Alizarin red staining analysis showed that the number of mineralized nodules was significantly reduced in the HG group (*p* < 0.001), indicating impaired osteoblast mineralization under high‐glucose conditions. Conversely, morroniside treatment significantly increased the number of mineralized nodules (*p* < 0.001), suggesting restoration of osteoblast mineralization capacity (Figure 3D). qRT‐PCR and Western blot analyses revealed that mRNA and protein expression of AGEs and RAGE was significantly upregulated under HG conditions, while morroniside treatment significantly downregulated these expressions (Figure 3E,F). DHE kit analysis of intracellular ROS generation showed that ROS production was significantly increased in HG conditions, an increase significantly reduced by morroniside treatment (Figure 3G). These findings suggest that morroniside may protect against DOP by inhibiting the AGEs‐RAGE axis and alleviating oxidative stress.

### 3.3 Reversal of AGE‐Induced Effects on Osteoblast Proliferation, Apoptosis, and Differentiation by Morroniside

**FIGURE 4:**
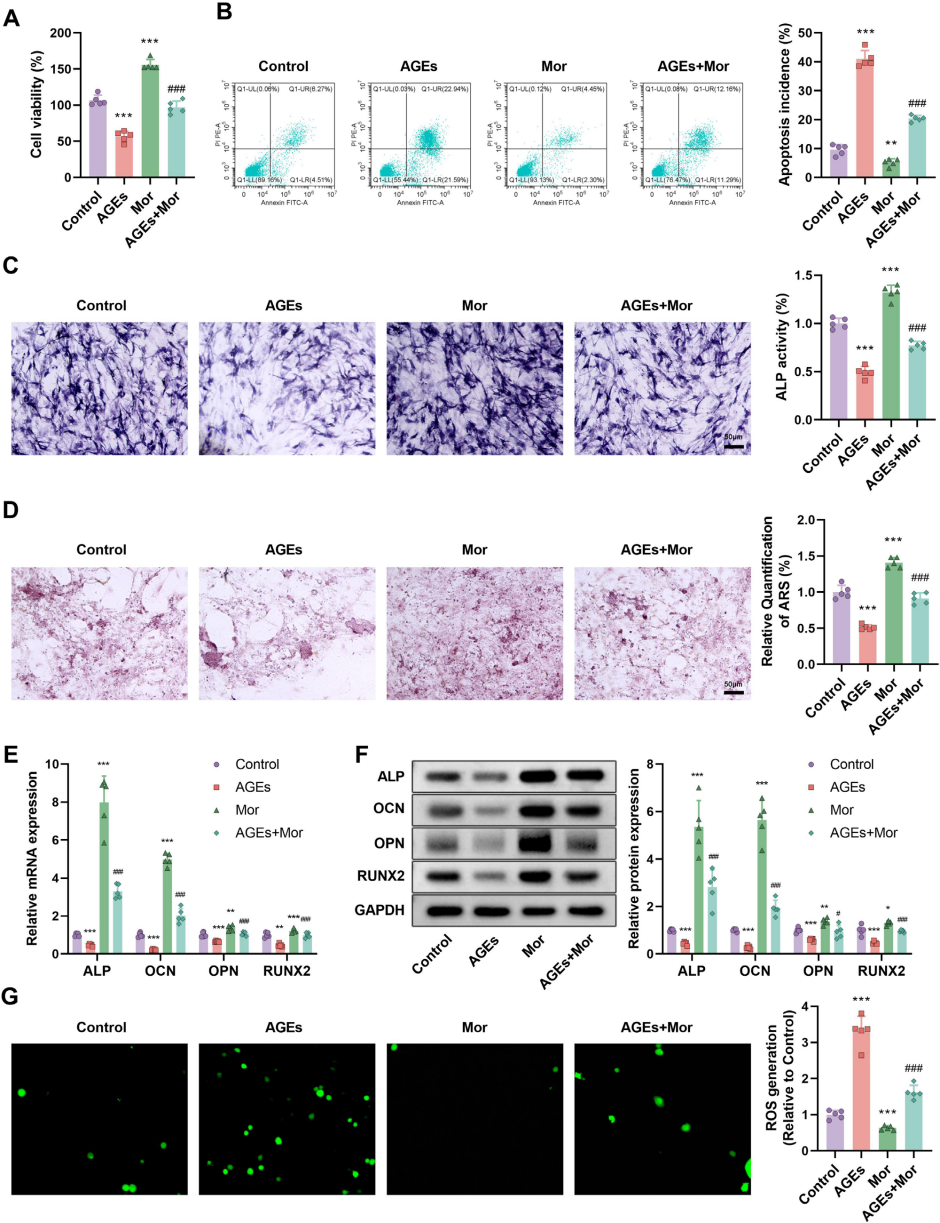
Reversal of AGEs‐induced effects on osteoblast proliferation, apoptosis, and differentiation by Morroniside. (A) MTT assay for cell viability and (B) Annexin V‐FITC/PI double staining for apoptosis in Control, AGEs, Morroniside, and AGEs + Morroniside groups. (C) ALP activity and (D) ARS quantification in the same groups. (E) mRNA and (F) Protein expression of osteogenic markers ALP, OPN, OCN, and RUNX2. (G) Intracellular ROS generation in the groups. *n* = 5, Data are presented as mean ± SEM. *p < 0.05, **p < 0.01, ***p < 0.001 versus Control; #*p* < 0.05, ##*p* < 0.01, ###*p* < 0.001 versus AGEs.

To investigate the reversal of AGE‐induced effects on osteoblast function by morroniside, we divided the experiment into four groups: the Control group, the AGEs group, the Morroniside (Mor) group, and the AGEs + Mor group. MTT assays measuring cell viability showed that AGEs treatment significantly reduced cell viability (*p* < 0.001), an effect that was significantly improved by morroniside treatment (Figure 4A). Annexin V‐FITC/PI double staining analysis indicated that AGEs treatment significantly increased apoptosis rates (*p* < 0.001), and this increase was significantly reduced by morroniside treatment, bringing the apoptosis rate in the AGEs + Mor group close to that of the Control group (Figure 4B). ALP activity assays and ARS quantification showed that AGEs treatment decreased ALP activity (Figure 4C, *p* < 0.001) and reduced ARS quantification (Figure 4D, *p* < 0.001). Morroniside treatment significantly increased ALP activity and ARS quantification. RT‐qPCR and Western blotting analyses of osteogenesis‐related markers ALP, OPN, OCN, and RUNX2 showed that mRNA and protein expressions of these genes were significantly reduced in the AGEs group (*p* < 0.001), while morroniside treatment significantly increased their expressions (Figure 4E,F). Fluorescence microscopy observations of intracellular ROS generation showed that AGEs treatment significantly increased ROS generation (Figure 4G, *p* < 0.001), and this increase was significantly reduced by morroniside treatment. These results suggest that morroniside may have potential therapeutic effects on AGE‐induced osteoporosis by reducing oxidative stress and regulating gene expression related to bone formation.

### 3.4 Wnt/β‐Catenin Pathway Involvement in Morroniside's Rescue of AGE‐Treated Osteoblast Differentiation Potential

**FIGURE 5:**
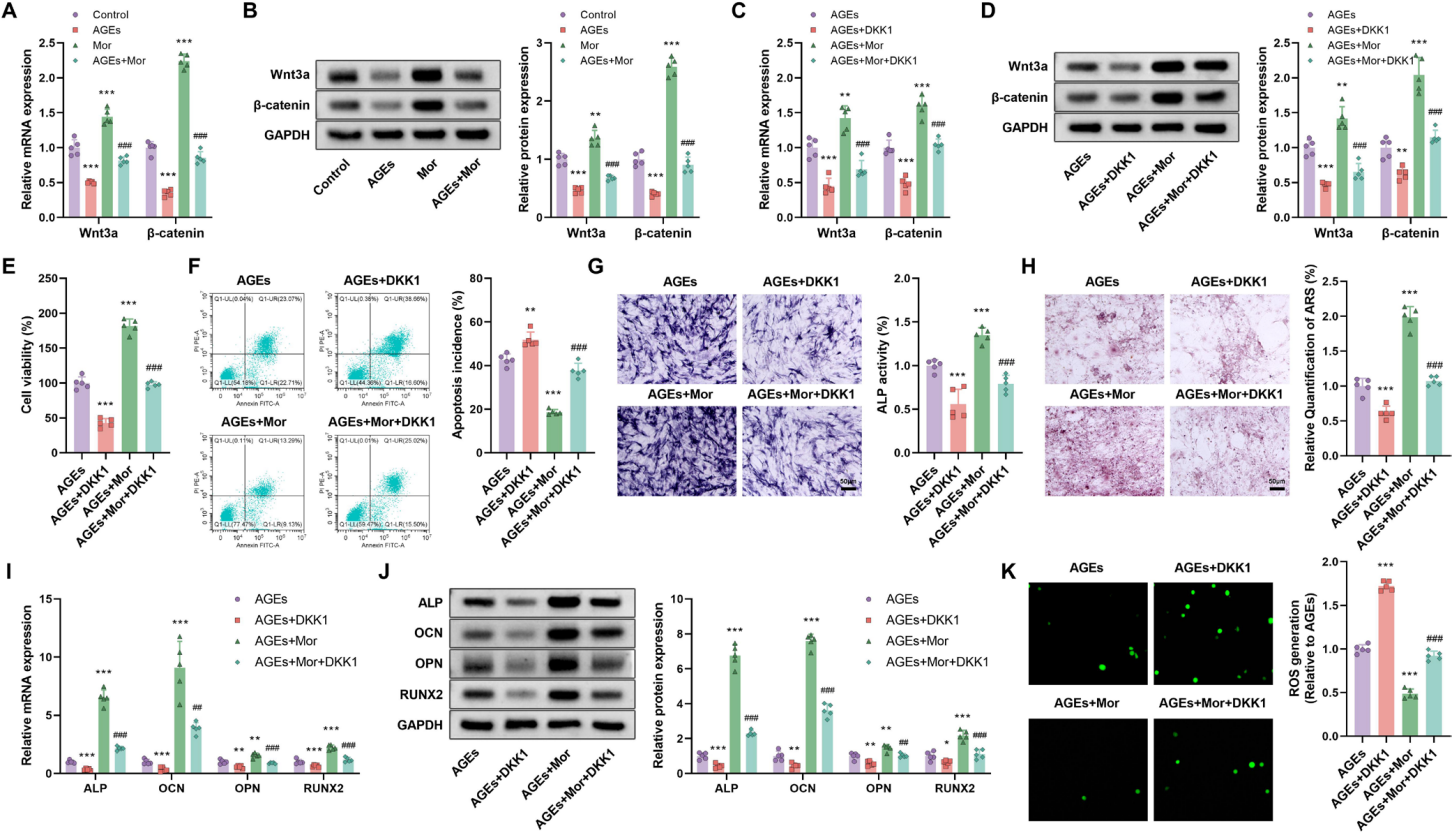
Involvement of the Wnt/β‐catenin signaling pathway in the protective effects of Morroniside on AGEs‐treated osteoblasts. (A and B) mRNA and protein expression of Wnt3a and β‐catenin in Control, AGEs, Morroniside, and AGEs + Morroniside groups. (C and D) Effects of DKK1 on Wnt3a and β‐catenin expression. (E–H) Cell viability, apoptosis rate, ALP activity, and ARS quantification in the presence of AGEs and/or DKK1 with or without Morroniside. (I and J) mRNA and protein expression of osteogenic markers ALP, OCN, OPN, and RUNX2. (K) ROS generation in the groups. *n* = 5, Data are presented as mean ± SEM. *p < 0.05, **p < 0.01, ***p < 0.001 versus Control; #*p* < 0.05, ##*p* < 0.01, ###*p* < 0.001 versus AGEs.

To explore the mechanisms underlying morroniside's effects, we examined the role of the Wnt/β‐catenin pathway. AGEs treatment significantly reduced mRNA and protein expression of Wnt3a and β‐catenin (*p* < 0.001), an effect partially reversed by morroniside treatment, bringing their expression levels closer to those of the Control group (Figure 5A,B). DKK1, an inhibitor of the Wnt pathway [20], significantly reduced mRNA and protein expression of Wnt3a and β‐catenin when added to the AGEs + DKK1 group, but morroniside treatment reversed this impact (Figure 5C,D). Cell viability and apoptosis rate assessments showed that the AGEs group had significantly lower cell viability and higher apoptosis rates compared with the Control group (Figure 5E,F), while morroniside treatment significantly increased cell viability and decreased apoptosis rates. The addition of DKK1 further reduced cell viability and increased apoptosis rates in the AGEs + DKK1 group, but co‐treatment with morroniside (AGEs + Mor + DKK1) partially restored these parameters. ALP activity and ARS quantification similarly showed that the AGEs group had lower ALP activity and reduced ARS quantification, while morroniside treatment significantly increased ALP activity and enhanced ARS quantification. The addition of DKK1 further decreased ALP activity and reduced ARS quantification in the AGEs + DKK1 group, but co‐treatment with morroniside (AGEs + Mor + DKK1) partially restored these parameters (Figure 5G,H). Gene expression analysis of bone formation‐related genes showed that mRNA and protein expressions of ALP, OCN, OPN, and RUNX2 were significantly lower in the AGEs group, but significantly increased by morroniside treatment. The addition of DKK1 further reduced these expressions in the AGEs + DKK1 group, but co‐treatment with morroniside (AGEs + Mor + DKK1) partially restored these parameters (Figure 5I,J). ROS generation analysis showed that ROS levels were higher in the AGEs group, but significantly reduced by morroniside treatment. The addition of DKK1 further increased ROS levels in the AGEs + DKK1 group, but co‐treatment with morroniside (AGEs + Mor + DKK1) partially reduced ROS levels (Figure 5K).

**FIGURE 6:**
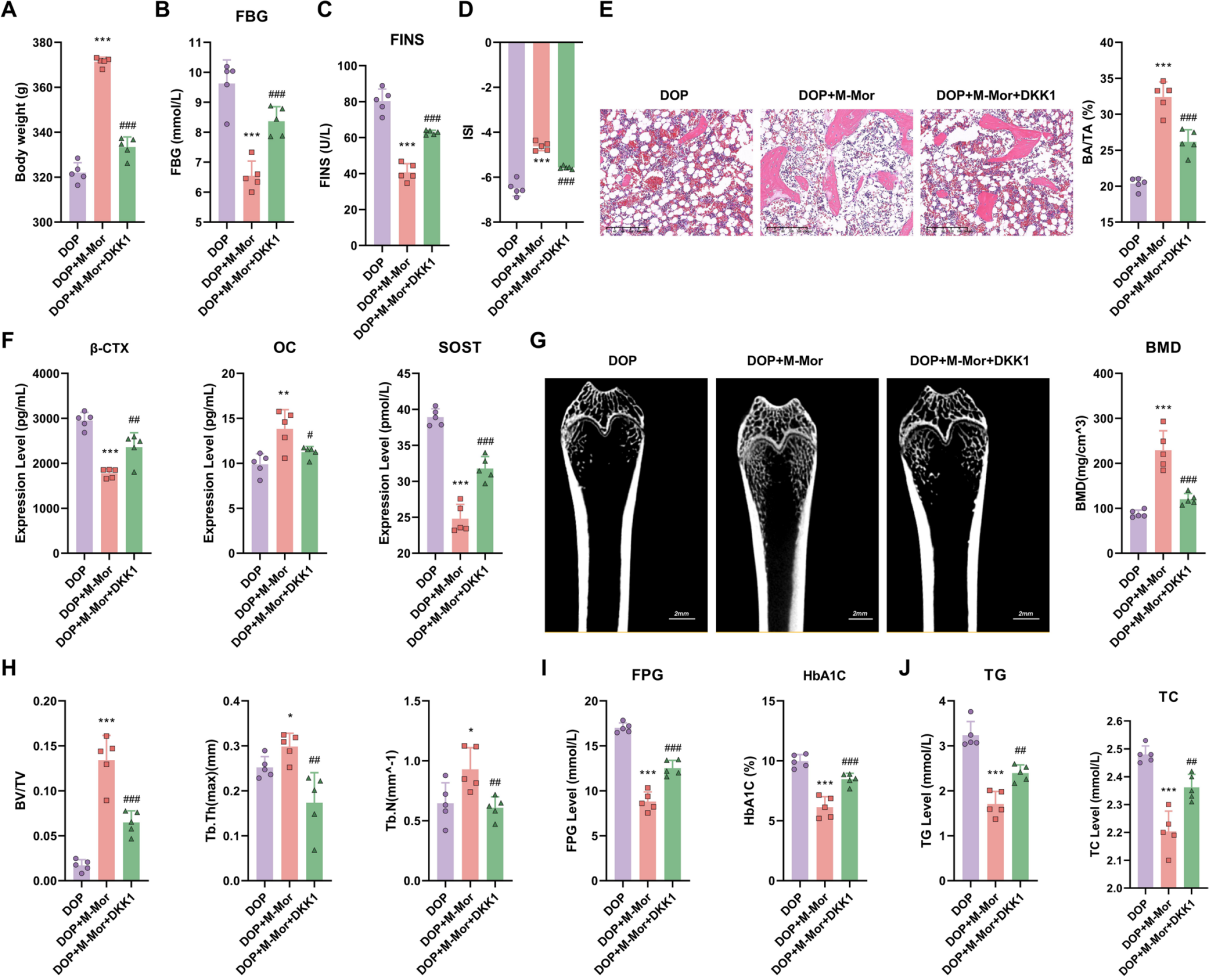
Mechanisms of Morroniside and its combination with DKK1 in a diabetic osteoporosis rat model. (A) Body weight, (B) Fasting blood glucose (FBG), (C) Insulin sensitivity (FINS), (D) Insulin sensitivity index (ISI), (E) Bone tissue structure, (F) Bone metabolism markers, (G) Micro‐CT images of the right femur in Control, DOP, DOP + L‐Mor, DOP + M‐Mor, and DOP + H‐Mor groups, (H) Measurement of trabecular bone parameters (bone volume fraction (BV/TV), trabecular thickness (Tb.Th), trabecular number (Tb.N)) in the right femur of each group, (I) Lipid metabolism indicators, and (J) Lipid levels in diabetic rats treated with Morroniside and/or DKK1. *n* = 5, Data are presented as mean ± SEM. *p < 0.05, **p < 0.01, ***p < 0.001 versus Control; #*p* < 0.05, ##*p* < 0.01, ###*p* < 0.001 versus DOP.

**FIGURE 7:**
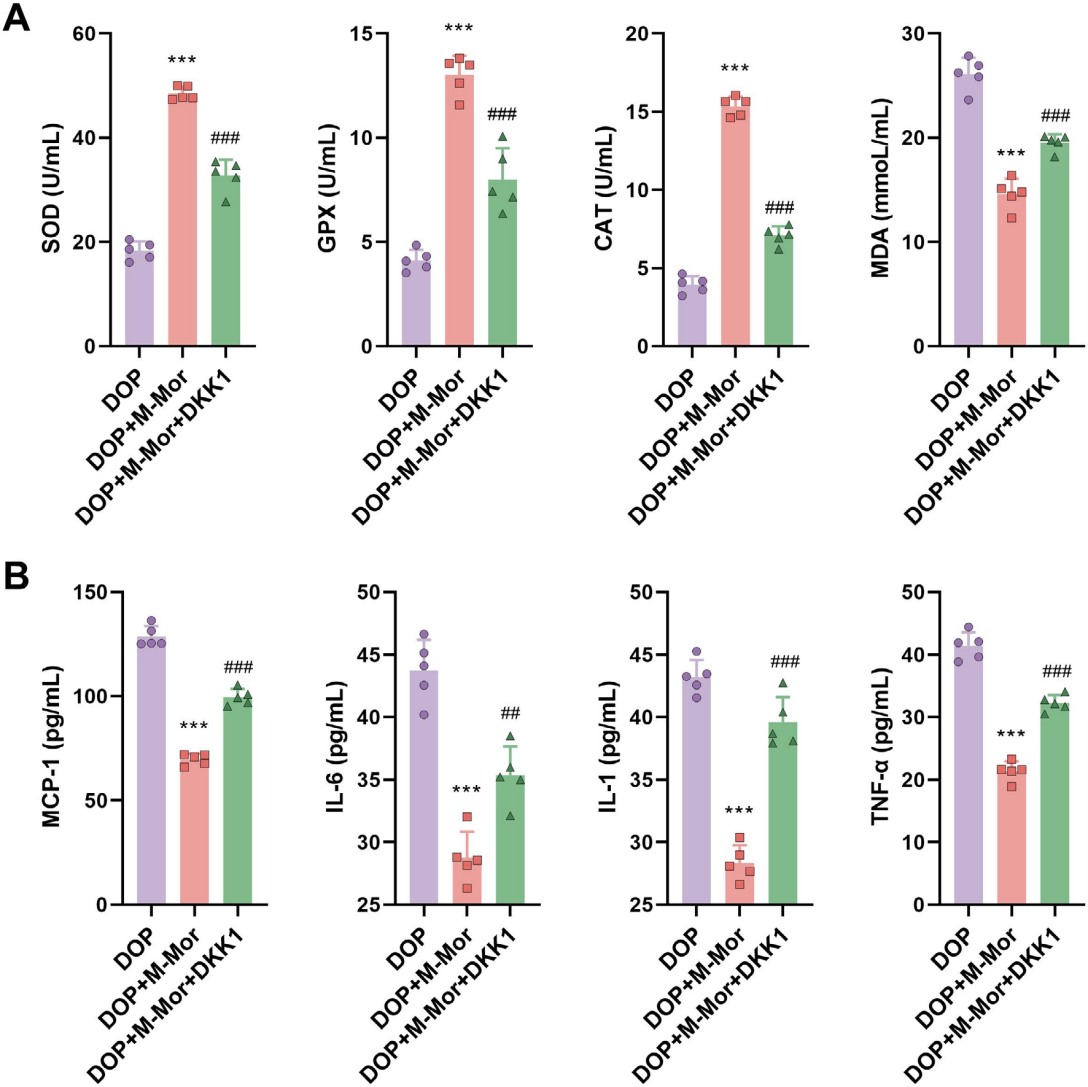
Serum oxidative stress and inflammation levels in diabetic rats treated with Morroniside and/or DKK1. (A) Activities of antioxidant enzymes (SOD, GPX, CAT) and malondialdehyde (MDA) levels, and (B) Inflammatory cytokine levels in Control, DOP, and Morroniside‐treated groups with or without DKK1. *n* = 5, Data are presented as mean ± SEM. *p < 0.05, **p < 0.01, ***p < 0.001 versus Control; #*p* < 0.05, ##*p* < 0.01, ###*p* < 0.001 versus DOP.

Further validation was conducted using a rat model of DOP. Experimental groups included the DOP control group, the DOP plus morroniside group (DOP + M‐Mor), and the DOP plus morroniside and DKK1 group (DOP + M‐Mor + DKK1). Comparisons between DOP + M‐Mor and DOP + M‐Mor + DKK1 highlighted the impact of DKK1 on the Wnt/β‐catenin pathway, thereby influencing the therapeutic efficacy of morroniside. Introduction of DKK1 (DOP + M‐Mor + DKK1) significantly inhibited the beneficial effects of morroniside, manifesting as attenuated improvements in body weight maintenance (Figure 6A), glycemic control (Figure 6B), insulin sensitivity (Figure 6C,D), bone structure quality (Figure 6E), bone metabolism marker expression (Figure 6F) and glucose and lipid levels (Figure 6I,J). Figure 6G presented micro‐CT images of bone structures for the DOP, DOP + M‐Mor, and DOP + M‐Mor + DKK1 groups, visually demonstrating differences in bone morphology and suggesting variations in bone micro‐architecture due to different treatments. For Figure 6H, measurements of BV/TV (bone volume/tissue volume), Tb.Th (trabecular thickness), and Tb.N (trabecular number) were shown. The DOP + M‐Mor group exhibited significant improvements in these parameters compared with the DOP group (as indicated by asterisks, suggesting statistical significance), while the DOP + M—Mor + DKK1 group showed intermediate or distinct values, highlighting the impact of the treatments on trabecular bone micro‐structure. Serum oxidative stress and inflammation levels also showed that DKK1 addition partially weakened the antioxidative effects of morroniside and attenuated its reduction of lipid peroxidation (Figure 7A). Serum inflammation levels further indicated that DKK1 partially compromised the anti‐inflammatory effects of morroniside (Figure 7B).

In summary, DKK1 exacerbated cellular damage under AGE conditions, whereas co‐treatment with morroniside partially reversed these negative effects. These findings suggest that morroniside may exert therapeutic effects on AGE‐induced osteoporosis by modulating the Wnt/β‐catenin signaling pathway.

## 4 Discussion

DOP is a critical complication of diabetes, characterized by reduced BMD and heightened fracture risk. Morroniside has been demonstrated to possess anti‐inflammatory, antioxidative, and metabolic regulatory properties [21]. In this study, we found that morroniside significantly alleviated weight loss, decreased FBG and fasting insulin (FINS) levels, and improved the insulin sensitivity index in diabetic rats. Additionally, it increased BMD and the percentage of bone area while reducing adipocyte numbers, indicating a beneficial effect on skeletal health in diabetic rats. These results align with previous studies, further supporting the potential of morroniside in treating diabetic complications. For example, morroniside exerts beneficial effects in regulating carbohydrate and lipid metabolism, improving glucose tolerance, and mediating antioxidant and anti‐inflammatory responses [22]. Studies have shown that in type 2 diabetic mice, morroniside significantly lowers serum AST and ALT levels, reduces liver inflammation activity scores, and protects against hepatic injury [23]. Moreover, it increases the number of pancreatic islets, ameliorates pancreatic damage, and enhances the antioxidant system to promote superoxide radical clearance [24].

The AGEs/RAGE signaling pathway plays a crucial role in diabetes and its complications [25]. AGEs (advanced glycation end‐products) are closely associated with diabetic complications, while RAGE (the receptor for AGEs) contributes to the development of diabetic vascular lesions when functionally aberrant. Chronic hyperglycemia in diabetes increases AGE formation, a process that can be counteracted by inhibiting RAGE receptors to prevent adverse effects on the vascular wall [26]. Wang et al. found that AGEs upregulate lysyl oxidase and endothelin‐1 expression in human aortic endothelial cells via the ERK1/2‐NF‐κB and JNK‐AP‐1 pathways, changes closely linked to diabetes progression and complications [27]. Mechanistic studies of the AGEs/RAGE signaling pathway in diabetic complications indicate that AGE‐mediated activation of RAGE triggers cellular signaling pathways involved in inflammatory responses and tissue damage [28]. Inhibition of RAGE receptors in diabetic NOD mice delays disease onset, highlighting the potential therapeutic value of targeting RAGE in diabetic complications [29]. Furthermore, AGEs reduce collagen cross‐linking, impairing osteoblast adhesion to the bone matrix, and inhibit alkaline phosphatase activity, disrupting bone mineralization [30]. Collectively, these factors contribute to the development of osteoporosis. Our study found that morroniside significantly reduces the expression of AGEs and RAGE, potentially alleviating oxidative stress and inflammatory responses to mitigate AGE‐induced damage to bone cells. This discovery provides direct evidence for the anti‐AGE effects of morroniside and underscores its potential application in the treatment of DOP.

Proliferation and differentiation of osteoblasts are critical processes in bone formation [31]. Studies show that osteoblasts participate in bone tissue formation and remodeling by secreting various growth factors and activating signaling pathways such as BMP2, Wnt, and Runx2 [32]. These factors not only promote bone tissue formation but also maintain skeletal health by regulating osteoblast survival and apoptosis. Our results indicate that morroniside promotes osteoblast proliferation, inhibits apoptosis, and enhances the expression of osteogenic differentiation markers. These findings highlight the significant role of morroniside in promoting skeletal health and repair, particularly under diabetic conditions. Related studies suggest that morroniside may inhibit apoptosis by influencing intracellular antioxidant pathways, such as Bcl‐2 expression [33]. In diabetic environments, osteoblast function is compromised: diabetes alters insulin‐like growth factor‐1 (IGF‐1) levels, which in turn impair osteoblast differentiation and proliferation [34]. Therefore, the role of morroniside in the diabetic setting is particularly important, as it helps maintain normal osteoblast function to promote skeletal health and repair.

Although this study primarily investigates the effects of morroniside on osteoblast proliferation, differentiation, and mineralization, DOP involves both impaired bone formation and enhanced bone resorption, the latter of which is mediated by osteoclasts. Morroniside's inhibition of AGE/RAGE signaling and reduction in inflammatory cytokines (e.g., TNF‐α, IL‐6) may indirectly suppress osteoclast activation, as AGEs and pro‐inflammatory factors are known to promote osteoclastogenesis through NF‐κB and MAPK pathways. However, the direct effect of morroniside on osteoclast differentiation and activity remains unaddressed in this study. Future research could explore the dual regulatory role of morroniside on both osteoblasts and osteoclasts, as well as the underlying molecular mechanisms involved in bone remodeling.

The Wnt/β‐catenin signaling pathway is central to regulating osteoblast differentiation [35]. Previous studies have identified the importance of the Wnt/β‐catenin pathway in bone development, osteogenesis, chondrogenesis, fracture healing, and various diseases [36, 37]. This pathway regulates osteoblast proliferation and differentiation through β‐catenin‐dependent mechanisms, with β‐catenin playing a core role in adult bone mass accumulation [38]. Activation of the Wnt signaling pathway promotes osteoblast differentiation and bone formation, with β‐catenin expression closely related to bone formation as a transcription factor in the Wnt signaling pathway [39]. In our study, morroniside significantly increased the expression of Wnt3a and β‐catenin, suggesting that it may enhance osteoblast differentiation and bone formation by activating the Wnt/β‐catenin signaling pathway. Co‐treatment with DKK1, an inhibitor of the Wnt pathway, diminished the promotive effect of morroniside on osteoblast differentiation, further confirming the central role of the Wnt/β‐catenin pathway in the action of morroniside. This demonstrates that activation of the Wnt/β‐catenin pathway is not an additional effect but a critical mechanism underpinning morroniside's protective role. While morroniside may exert pleiotropic effects, such as inhibiting AGE/RAGE signaling and reducing oxidative stress, the dependency on Wnt/β‐catenin pathway activation—evidenced by the DKK1‐mediated reversal—highlights its essential role in promoting osteoblast function and bone formation. These findings align with the known importance of Wnt/β‐catenin signaling in bone homeostasis, where its suppression contributes to impaired osteogenesis in DOP. Future studies could explore potential crosstalk between the Wnt/β‐catenin pathway and other signaling networks modulated by morroniside.

Although the current study used a DOP rat model, which may differ from the pathophysiological processes in clinical patients, and the optimal therapeutic dose and specific mechanisms of M‐Mor require further investigation, our research remains innovative and clinically significant. The innovation lies in not only validating the therapeutic effects of morroniside on DOP but also exploring its molecular mechanisms in depth. Specifically, we revealed that morroniside improves osteoblast function and bone health by inhibiting AGE/RAGE signaling and activating the Wnt/β‐catenin pathway. This finding not only provides scientific support for the clinical application of morroniside but also offers new targets for DOP treatment.

## Conflicts of Interest

The authors declare no conflicts of interest.

## Data Availability

The data that support the findings of this study are available from the corresponding author upon reasonable request.
